# Brain Routes for Reading in Adults with and without Autism: EMEG Evidence

**DOI:** 10.1007/s10803-013-1858-z

**Published:** 2013-06-09

**Authors:** Rachel L. Moseley, Friedemann Pulvermüller, Bettina Mohr, Michael V. Lombardo, Simon Baron-Cohen, Yury Shtyrov

**Affiliations:** 1 MRC Cognition and Brain Sciences Unit, 15 Chaucer Road, Cambridge, CB2 7EF UK; 2 Brain Language Laboratory, Department of Philosophy and Humanities, Freie Universität, Berlin, Germany; 3 Anglia Ruskin University, Cambridge, UK; 4 Department of Psychiatry, Autism Research Centre, University of Cambridge, Cambridge, UK; 5 Centre for Functionally Integrative Neuroscience, Aarhus University, Aarhus, Denmark; 6 Centre for Languages and Literature, Lund University, Lund, Sweden

**Keywords:** Reading, Dual-route model, Hyperlexia, Semantics, EEG, MEG

## Abstract

Reading utilises at least two neural pathways. The temporal lexical route visually maps whole words to their lexical entries, whilst the nonlexical route decodes words phonologically via parietal cortex. Readers typically employ the lexical route for familiar words, but poor comprehension plus precocity at mechanically ‘sounding out’ words suggests that differences might exist in autism. Combined MEG/EEG recordings of adults with autistic spectrum conditions (ASC) and controls while reading revealed preferential recruitment of temporal areas in controls and additional parietal recruitment in ASC. Furthermore, a lack of differences between semantic word categories was consistent with previous suggestion that people with ASC may lack a ‘default’ lexical-semantic processing mode. These results are discussed with reference to dual-route models of reading.

**Electronic supplementary material:**

The online version of this article (doi:10.1007/s10803-013-1858-z) contains supplementary material, which is available to authorized users.

## Introduction

Despite multiple conceptual reformations since Kanner’s (1943) classic autism description, language/communication abnormalities and impairments have remained a cornerstone of the diagnosis of autism spectrum conditions (ASC). Within the auditory domain, children and adults with ASC lack the neural preference and behavioural inclination towards speech sounds typically present from a very early age (Klin 1991; Kuhl et al. 2005; Muller 2007; Groen et al. 2008; Lai et al. 2012). Though this might be secondary to a broader failure in *social* orientation (Rapin 1997; Dawson et al. 1998; Swettenham et al. 1998; Schultz et al. 2000), it would appear to be independent of sensory deficits and has been argued to be specific to human speech (Čeponiené et al. 2003). These studies suggest, therefore, that linguistic stimuli may be treated in a qualitatively different way within the autistic brain.

This is equally true in the visual domain, though the processing of written words in ASC has received less attention. Gaffrey et al. (2007), in an fMRI task of semantic decision, discovered unusually elevated recruitment of visual cortex (striate and extrastriate areas, BA 17, 18, 19). Similar strong recruitment of extrastriate cortex during a sentence processing task was reported by Kana et al. (2006). Since lower activity in BA 45 during semantic processing has also been reported (Harris et al. 2006), several authors have suggested a qualitatively different strategy for lexicosemantic processing in autism (Kamio and Toichi 2000; Toichi and Kamio 2001, 2002, 2003; Gaffrey et al. 2007): one that, somewhat immature, relies excessively on visualisation and perceptual processing at the expense of deep semantic analysis of the visually- or verbally-presented linguistic material.

Järvinen-Pasley et al. (2008) commented that, in autism, “semantic-level processing is not the primary, or ‘default’ speech processing mode” (pp. 117). Indeed, processing the semantic rather than surface visual features of words does not lead to stronger recall in people with autism, unlike in the typical population (the ‘levels of processing’ effect: Toichi and Kamio 2002; Harris et al. 2006; Lombardo et al. 2007). Furthermore, they do not benefit from semantic cues in recall (Mottron et al. 2001) or semantic primes in decision tasks (Kamio et al. 2007), and fail to use semantic chunking strategies during processing (Hermelin and O’Connor 1970). This might explain why semantic processing abnormalities and subtle impairments are considered a hallmark of ASC by many authors (Harris et al. 2006; Walenski et al. 2006; Gaffrey et al. 2007; Braeutigam et al. 2008).

These abnormalities may contribute to difficulties with reading comprehension that are revealed by lower scores in standardised batteries (Venter et al. 1992; Myles et al. 2002; Nation et al. 2006; Newman et al. 2007). When reading text and phrases, several studies reported that participants with ASC fail to utilise semantic context to infer sometimes ambiguous meaning (Frith and Snowling 1983; Happé 1997; Wahlberg and Magliano 2004), make errors that are semantically inappropriate (though syntactically correct) when filling blank spaces (Frith and Snowling 1983; Snowling and Frith 1986), and have difficulty answering questions based on passages (O’Connor and Hermelin 1994). These reports suggest that individuals with autism might have difficulty reading for meaning and/or in activating semantic processes, particularly, as in the words of Järvinen-Pasley et al. (2008), when not explicitly asked to do so (though, consistent with greater strengths in perceptual processing, they can benefit from implicit semantic cues presented pictorially [Kamio and Toichi 2000; Sahyoun et al. 2010]).

In contrast, the previous literature suggests that the typical population show semantic activation related to sensorimotor associations of words even without explicit processing instructions or focused attention—suggesting automatic activation of neural circuits representing word meaning (Pulvermüller et al. 2005; Pulvermüller and Shtyrov 2006; González et al. 2006; Hauk et al. 2008; Kiefer et al. 2008; Shtyrov et al. 2004, 2010; Barrós-Loscertales et al. 2011). In typical individuals, this activity reflects differential brain topographies for the representation of words with different meanings. Action words, for example, have been strongly associated with the cortical motor system, even in a specifically somatotopic manner that reflects their association with the effectors of the body (Hauk et al. 2004, 2008; Pulvermüller et al. 2005; Tettamanti et al. 2005; Aziz-Zadeh and Damasio 2008; Kemmerer et al. 2008). In contrast, words for objects with strongly visual associations evoke activity in the temporo-occipital object processing stream (Warburton et al. 1996; Pulvermüller et al. 1999; Martin and Chao 2001; Martin 2007). Since people tend to learn the word for an action or object in the context of experiencing/interacting with it, such organisation is proposed to arise through Hebbian principles due to the simultaneous activation of sensorimotor perceptual regions and core perisylvian language cortex (Pulvermüller 2001). Consequently, word phonology, articulatory features and meaning are represented at a brain level in distributed neuronal assemblies reaching into action and perception parts of the brain (“action-perception circuits”: see Pulvermüller and Fadiga 2010).

What happens in the brain when people read written words? One theory suggests that there are two neural routes through which written symbols on the page are transformed into meaningful units (Coltheart et al. 2001). In one strategy, whole visual word-forms are mapped directly onto their corresponding lexical entries, thus transparently matching symbol to meaning. This lexicosemantic route, otherwise known as the ‘direct’ pathway from word to meaning (McCarthy and Warrington 1986; Coltheart et al. 2001), is associated with a ventral pathway, which involves activation of left-hemispheric occipito-temporal areas such as the fusiform gyrus (Fiebach et al. 2002; Jobard et al. 2003; Levy et al. 2009), typically implicated in visual word-processing (Cohen et al. 2002; Kronbichler et al. 2004). In contrast, a dorsal pathway processes written symbols in a piecemeal manner, converting graphemes to their auditory phoneme counterparts, which can then be spoken aloud or further processed for meaning via their pronunciations. This grapheme-phoneme conversion (or nonlexical) route is associated with left parietal cortex, including superior parietal lobule, inferior parietal and supramarginal gyrus, and also pars opercularis (Fiez et al. 1999; Jobard et al. 2003; Mechelli et al. 2003; Levy et al. 2009), known to be involved in general phonological processing (Paulesu et al. 1993; Fiez 1997; Poldrack et al. 1999; McDermott et al. 2003). The existence of dorsal and ventral routes for language processing has been equally supported in the auditory domain, where, like visual letters, sounds are mapped to articulation via the dorsal connections of the arcuate and superior longitudinal fasciculus; higher-level meaning comprehension is served by the extreme capsule in a ventral stream linking temporal to inferior frontal structures (Saur et al. 2008).

Whilst skilled readers may utilise and shift between either pathway, modulated by features of the written words such as frequency, transparency and orthographic regularity (Zevin and Balota 2000), there is evidence that highly frequent, familiar words are preferentially processed directly via the lexicosemantic route in a holistic fashion (Coltheart and Rastle 1994). However, the aforementioned problems with word comprehension would suggest that the same may not be true in autism.

Interestingly, this difficulty often presents in conjunction with hyperlexia (Healy et al. 1982; Whitehouse and Harris 1984; Goldberg 1987; Smith and Bryson 1988; Patti and Lupinetti 1993; O’Connor and Hermelin 1994; Grigorenko et al. 2002; Newman et al. 2007), which early accounts defined as a “compulsion to decode written material without comprehension of its meaning” (Whitehouse and Harris 1984) but which is also often defined as being able to read before the age of starting school. Compulsive hyperlexia has also been observed in stroke patients as a “release phenomenon” following brain damage (Berthier et al. 2006). In autism, this decoding skill possesses a savant-like quality, generally far outstripping reading comprehension: along with the ability to read novel pseudowords (Frith and Snowling 1983; Nation et al. 2006; Newman et al. 2007), this suggests the integrity of the grapheme-phoneme conversion route in autism, and that this route is perhaps enhanced and relied upon rather than whole-word matching (Aram et al. 1984; Goldberg and Rothermel 1984; Aram and Healy 1988). However, neuroscientific evidence for an over-emphasis on asemantic reading in ASC, even for familiar words, is still not available.

In order to investigate the neural routes for visual word-processing in the autistic brain, we used combined electroencephalography and magnetoencephalography (EEG/MEG or EMEG) to compare the time-course and localisation of brain activity in subjects with an ASC with typical controls. A passive reading task was employed to investigate pathways activated by reading short, simple words. A passive perceptual paradigm has previously been used in the typical population to investigate the processing of different semantic categories, which evoke differential patterns of neural activity (Hauk et al. 2004; González et al. 2006; Barrós-Loscertales et al. 2011), even at early latencies and without conscious attention (Shtyrov et al. 2004; Pulvermüller et al. 2001, 2005; Moscoso del Prado Martin et al. 2006; Hauk et al. 2008). Given the aforementioned literature on reading comprehension and semantic processing in ASC, it is unclear whether the same is true in ASC. As the present experiment involved the same passive reading paradigm with words of different semantic meaning, we therefore also decided to look at differences between word categories within our stimulus set, in order to investigate whether semantic category-specific differences also arise automatically in ASC as they do in the typical population.

## Methods

### Participants

14 Participants with high-functioning ASC (13 with Aspergers’ Syndrome, 1 with PDD-NOS) and 17 typically-developed control participants took part in the study, all monolingual native speakers of English. The groups were matched for full-scale IQ as measured by the Cattell Culture Fair test (Cattell and Cattell 1960) (115.8 for controls and 118.5 for ASC respectively: t [29] = .389, *p* < .700), and with 11 males in the control group and 7 in the ASC group, contained a roughly equal division of sex ratio, with no significant difference in this (t [29] = .808, *p* < .430). Both groups were right-handed, though scores on the Edinburgh Handedness Inventory (Oldfield 1971) indicated that the ASC group were slightly less strongly lateralised (t [29] = 2.249, *p* = .032).

Eligibility for the study required that all ASC participants had received a formal clinical diagnosis using DSM-IV criteria. On the Autism Spectrum Quotient (AQ: Baron-Cohen et al. 2001), they scored significantly higher (37.3 ± SD9.9) than did the control group (13.8 ± 5.7; t [29] = 8.126, *p* < .001), indicating a significantly greater number of autistic traits. It was not possible to fully match the mean age of the groups, with the ASC group being slightly older than controls (31.4 ± 8.2 years vs. 25.0 ± 5.1 years; t [29] = 2.638, *p* < .014).

### Materials

The study employed an extensive corpus of 360 words matched for length, letter bigram and trigram frequency and number of orthographic neighbours, along with 120 length-matched hash-mark strings that acted as a low-level visual control condition. These psycholinguistic properties were retrieved from the CELEX database (Baayen et al. 1993). Prior to the EMEG experiment, a semantic rating study was performed by a group of 10 native English speakers (see Pulvermüller et al. 1999, for procedural details) in order to obtain participant ratings for each word on a number of semantic variables, including sensorimotor features (imageability, concreteness and action-relatedness) and affective-emotional features (arousal and valence). In accordance with these semantic ratings, the 360 experimental words consisted of 120 action-related (e.g. “knead”, “jog”), 120 object-related (e.g. “hawk”, “cheese”), and 120 abstract (e.g. “faze”, “fluke”) words which were used here as fillers. Naturally, due to their semantic associations, these word categories differed in action-relatedness, imageability, and other semantic variables: please see Online Resource 1 for details of their psycholinguistic and semantic properties.

#### Procedure

Having given informed consent, participants completed the Cattell Culture Fair test (Cattell and Cattell 1960), the Edinburgh Handedness Inventory (Oldfield 1971) and the AQ (Baron-Cohen et al. 2001) prior to EMEG preparation. Once prepared for the recording, participants were made comfortable and requested to stay as still as possible, avoiding all unnecessary movements, and to focus on a central fixation point whilst attending to the stimuli appearing on the screen. The experimental task, split into three blocks of approximately 7 min each, involved passive reading of the experimental stimuli which were presented tachistoscopically for 150 ms, in light grey font on a black background, with an inter-stimulus interval of 2,500 ms. So as to avoid order effects, two pseudo-randomised stimulus lists were counterbalanced between subjects in both groups. Between each 7 min block of the experimental task, participants were given a couple of moments to rest if required.

Following the experimental procedure to check attendance to the task, participants were given an unseen word recognition test containing a combination of 50 experimental and 25 novel distractor words chosen from a bank of length-matched words which did not make the final stimulus selection. No differences in performance emerged between the two groups (t [29] = 1.721, *p* < .110) and both performed above chance (average hit rate for controls: 82 ± 8.6 %; average hit rate for ASC: 74 ± 14.8 %).

#### EMEG Recording and Data Pre-processing

Electroencephalogram (EEG) and magnetoencephalogram (MEG) were simultaneously recorded in a magnetically- and acoustically-shielded MEG booth (IMEDCO Corp, Switzerland). EEG was recorded from electrode caps (EasyCap, Falk Minow Services, Herrsching-Breitbrunn, Germany) with 70 Ag/AgCl electrodes arranged according to the extended 10/10 % system. For MEG, the study employed a whole-head 306-channel MEG setup of 204 planar gradiometers and 102 magnetometers (Elekta Neuromag, Helsinki, Finland), which continuously recorded magnetic fields and field gradients during the task. Head position was tracked throughout the session using 5 magnetic coils, attached to the EEG cap, whose position with respect to three standardised points (nasion, left and right pre-auricular points) was digitised using the Polhemus Isotrak digital tracker system (Polhemus, Colchester, VT, USA). Further anatomical co-registration with MRI scans was made possible through additional digitisation of EEG electrodes and randomised points distributed over the scalp. In order to reject trials disturbed by blinking or eye saccades, eye movements were monitored by four EOG electrodes placed laterally to each eye (horizontal EOG) and vertically above and below the left eye (vertical EOG).

**Fig. 1:**
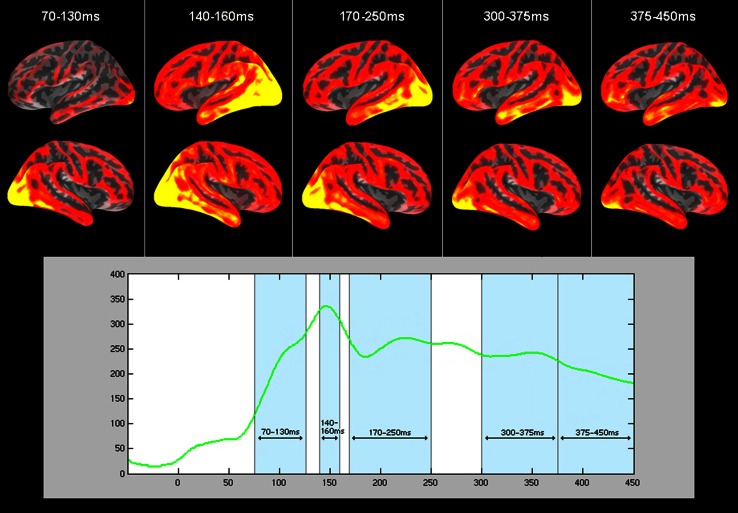
GLOBAL signal-to-noise ratio (SNR, for all subjects pooled) curve for all words during the 500 ms epoch, and activation for all words depicted within the five time-windows of focus. For the source estimations, activity in the* left* (*top*) and* right* (*middle*) hemispheres has been pooled for both subject groups in each time-window

Recordings were preprocessed offline using MaxFilter software (Elekta Neuromag, Helsinki), which employs the Signal-Space Separation method (Taulu and Kajola 2005; Taulu et al. 2004) to minimise external noise and sensor artefacts, along with spatio-temporal filtering and head-movement compensation to correct for between-block movements; any bad EEG/MEG channels were identified and re-interpolated. The MNE 2.7 software package (A. Martinos Center for Biomedical Imaging, Charlestown, MA, USA) was used throughout the rest of the analysis. Data were band-pass filtered between 0.1 and 30 Hz. For averaging, epochs of 500 ms were taken from 50 ms prior to stimulus onset: for baseline correction, mean amplitude over this 50-ms interval was later subtracted from the signal at all time-points. Epochs with an amplitude exceeding 150 μV in EEG and EOG channels and 2,000 fT/cm and 3,500 fT in gradiometer and magnetometer channels respectively were discarded, and remaining epochs were averaged within individuals for each stimulus type. For an unbiased estimate of the overall neural dynamics in response to verbal stimuli, a global signal-to-noise ratio (SNR) was calculated for all participants pooled by dividing amplitude at each time-point by the standard deviation in the baseline period (the first 50 ms) and then computing the root-mean square of SNR across all sensors. Peaks and troughs on this global SNR curve, averaged across all participants (Fig. 1), were identified, and these time periods were subjected to further source reconstruction and statistical analysis.

#### MRI Acquisition and EMEG Source Reconstruction

In order to explore the neuronal generators underlying electrophysiological and neuromagnetic activity, L2 minimum norm source estimations (Hämäläinen and Ilmoniemi 1994) for combined EEG/MEG data were computed using MNE and Freesurfer 4.3 software (Martinos Centre for Biomedical Imaging) in conjunction with individual subject structural MRI images used to model cortical grey matter surface. High-resolution structural T1 scans for each subject were acquired with a 3T Siemens Tim Trio MRI scanner (parameters of the MPRAGE sequence were as follows: field-of-view 256 mm × 240 mm × 160 mm, matrix dimensions 256 × 240 × 160, 1 mm isotropic resolution, TR = 2,250 ms, T1 = 900 ms, TE = 2.99 ms, flip angle 9°). They were preprocessed and coordinates aligned to EMEG data using digitised positions of the anatomical landmarks, electrodes and the head surface. A 3-shell boundary-element model for each subject, using inner and outer skull and skin surfaces, was created using a watershed algorithm. Source estimates for each stimulus type were computed for each subject and then morphed to the average brain (averaged from all subjects pooled), and grand averages for control and ASC groups were then computed to be displayed on the inflated average cortical surface.

Source activations for words compared with control condition in the grand averages calculated for both groups were statistically explored in a regions-of-interest (ROI) approach. Secondly, category-specific differences between the broken-down categories of action, object and abstract words were investigated. ROIs were anatomically defined based on the Desikan-Killiany Atlas subdivisions of the brain (Desikan et al. 2006) as implemented in the Freesurfer package. We then analysed source dynamics in those lobes of the brain where reading-related activity can be expected, namely occipital, parietal, temporal and frontal lobes, which included the following structures: (1) frontal cortices (covering superior frontal, middle frontal dorsal, middle frontal ventral, caudal frontal, BA 47, BA 45, BA 44, precentral, paracentral), (2) temporal cortices (superior temporal, middle temporal, inferior temporal, fusiform), (3) parietal cortices (postcentral, supramarginal, superior parietal, inferior parietal), and (4) occipital cortices (BA 17, BA 18 dorsal, BA 18 ventral, BA 19 dorsal, BA 19 ventral). Please see Online Resource 2 for a depiction of regions. Both left-hemispheric cortices and their right-hemisphere homologues were analysed. Where differences appeared, individual regions were further explored. In this more detailed analysis, three large regions (middle frontal cortex, precentral strip and occipital cortex) were subdivided into dorsal–ventral portions in accordance with the same anatomical guide, in order to assess more fine-grained group differences. Amplitudes of the source currents within these lobes/ROIs were calculated in the time-windows of interest defined through inspection of the SNR curve, as described above.

With all statistical analysis, Huynh–Feldt correction was applied to correct for sphericity violations wherever appropriate. Corrected *p* values are reported throughout.

## Results

Visual inspection of the global SNR curve revealed several peaks and windows for focus (see Fig. 1). The signal demonstrated a sharp increase from ~70 ms onwards with a peak around 150 ms followed by a downstroke and a plateau. We therefore analysed (1) the upstroke period between 70 and 130 ms, (2) the peak interval at 140–160 ms, and (3) the decline of this peak and the start of the following plateau at 170–250 ms. We also studied later periods of the epoch, capturing the wave between 300 and 375 ms, and the final stretch, between 375 and 450 ms, given the previous literature on lexical and semantic effects in M350 (Embick et al. 2001; Pylkkanen and Marantz 2003) and N400 (Kutas and Federmeier 2011; Lau et al. 2008) time ranges.

As can be seen in the source estimations in Fig. 1, written word stimuli evoked widespread activity across visual areas and perisylvian language regions, including the length of the temporal cortex and the inferior frontal gyrus, alongside additional motor, parietal and frontal activity. With the exception of the first time-window, activity in these regions appears slightly stronger and more widespread in the left hemisphere. Initially, within the 70–130 time-window where brain responses first differentiate between groups (see below), the majority of activity occurred in primary visual cortex, though activation also presents in inferior frontal cortex. Activity was seen to spread in an anterior fashion along the temporal cortex, increasing in the temporal pole and decreasing in posterior temporal regions by the late time-windows in 300–450 ms range.

### General Reading with Different Pathways

**Fig. 2:**
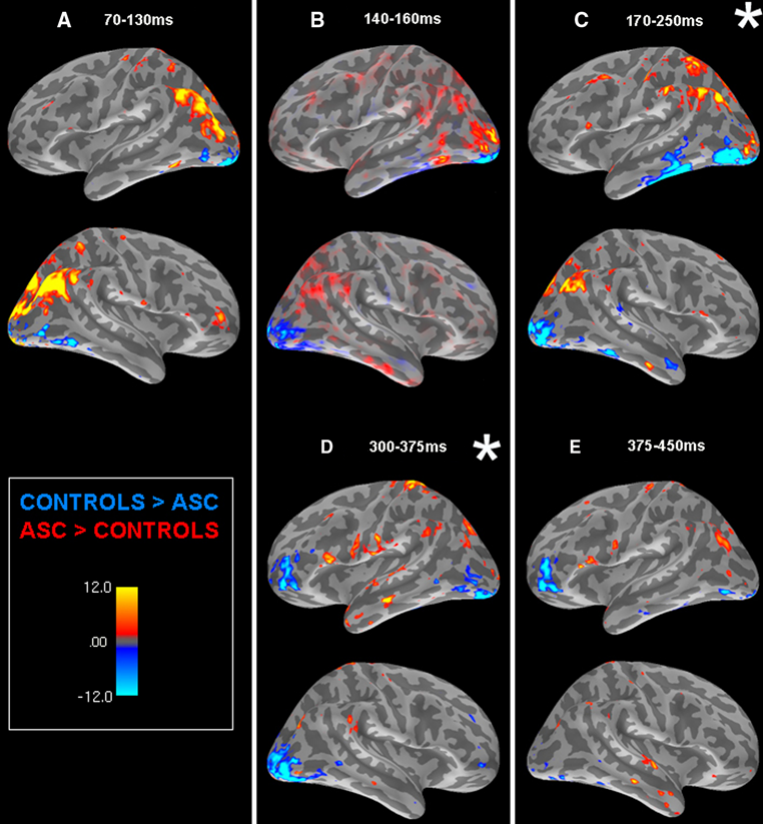
SOURCE estimations reflect contrasts between the two groups: areas of greater activity for control than ASC participants in *blue*, areas of greater activity for ASC than control participants in *red*. Source estimates are averaged across each time-window of focus. Time-windows in which group differences were significant specifically during word but not hash-mark reading are marked by an *asterisk* (*)

An initial ROI analysis focused on differences in brain activation between groups in these time-windows (Fig. 2). In each time-window independently, an ANOVA was conducted to examine activity in each lobe (frontal, temporal, parietal and occipital), each of which was split into a number of individual ROIs (see Methods for details). Where group differences were indicated within lobes (i.e. at the level of ROIs), these within-lobe regions were explored in ANOVAs including the level of Group (2) and ROI. These ANOVAs were run independently in each time-window for both the word-reading and the hash-mark condition, but the results discussed below are for the word-reading condition unless explicitly stated. Areas where main effects of group arose in each time-window are reported in Table 1, bolded for results which were specific for the word-reading condition. All results are summarised below.

**Table 1:** Group differences for word-reading in each time-window

	Main effects of group		Interactions
	Control > ASC	ASC > Control	
70–130 ms	No main effects of group	Bilateral superior parietal cortex (f [1, 29] = 5.511, *p* < .03) Bilateral inferior parietal cortex ([1, 29] = 8.601, *p* < .01) Bilateral dorsal BA 19 (f [1, 29] = 2.266, *p* < .03) Bilateral precentral* and paracentral gyrus (f [1, 29] **=** **4.471,** ***p*** **<** **.05)** * **t-tests of segmented precentral regions:** **Dorsal (t [29]** **=** **2.350,** ***p*** **<** **.03)** **Middle (t [29]** **=** **2.279,** ***p*** **<** **.03)**	No interactions
140–160 ms	No main effects of group	No main effects of group	**Bilateral parietal cortex: ROI (4)** **×** **hemisphere (2)** **×** **Group: f [3, 87]** **=** **2.843,** ***p*** **<** **.05**
170–250 ms	**Left temporal cortex (f [1, 29]** **=** **4.590,** ***p*** **<** **.05)** **L. inferior temporal gyrus (t [29]** **=** **2.793,** ***p*** **<** **.01)** ***L. fusiform gyrus (t [29]*** **=** ***1.944, p*** **<** ***.065)***	**Left parietal cortex (f [1, 29]** **=** **4.550,** ***p*** **<** **.04)** **L. supramarginal gyrus (t [29]** **=** **2.173,** ***p*** **<** **.04)** **L. superior parietal cortex (t [29]** **=** **2.244,** ***p*** **<** **.04)** ***L. inferior parietal cortex (t [29]*** **=** ***1.902, p*** **<** ***.07)***	**Left parietal and temporal cortices:** **Lobe (2)** **×** **Group: f [1, 29]** **=** **12.905,** ***p*** **<** **.005** **Lobe (2)** **×** **ROI (4)** **×** **Group: f [3, 87]** **=** **4.883,** ***p*** **<** **.01**
300–375 ms	No main effects of group	**Left parietal cortex (f [1, 29]** **=** **5.081,** ***p*** **<** **.04)** **L. pars opercularis (BA 44) (BA 44: t [29]** **=** **2.211,** ***p*** **<** **.04)** Precentral* and paracentral gyrus (f [29] **=** **4.249,** ***p*** **<** **.05)** * **t-tests of segmented precentral regions:** **Dorsal (t [29]** **=** **2.771,** ***p*** **<** **.02)** **Middle (t [29]** **=** **2.612,** ***p*** **<** **.02)**	**Left frontal lobe:** **ROI (9)** **×** **Group: f [8, 232]** **=** **2.813,** ***p*** **<** **.005**
375–450 ms	No main effects of group	No main effects of group	**Left frontal lobe: ROI (9)** **×** **Group: f [8, 232]** **=** **3.261,** ***p*** **<** **.04**

Significant interactions and main effects of group found in each time-window whilst reading. The second column indicates areas where control participants showed greater activation than individuals with ASC, whilst the third column reflects the opposite pattern. Bold text indicates interactions and group differences which were specific for words in that particular region and which did not appear for the hash-mark condition. Marginally non-significant effects are shown in italicised font, bolded where they refer specifically to the word-reading condition

Overall, the most striking observation of the analysis was a contrast between subject groups during word reading in which control participants showed a ventral spread of activation whilst those with ASC exhibited activation of the dorsal parietal route. As can be seen in Fig. 2 (Part A) and Table 1, the dorsal trend for the ASC group was predominantly non-specific for words until the peak of the SNR curve (140–160 ms: see Fig. 2, Part B), where an interaction of ROI, hemisphere and group was driven by greater activity in parietal cortex for the ASC group. The ASC group also showed greater word-specific activity than controls in left parietal cortex in the 170–250 ms (see Fig. 2, Part C) and 300–375 ms (see Fig. 2, Part D) time-windows. This greater word-specific activity in the latter window also included other parts of the dorsal pathway for phonological processing, namely pars opercularis (BA 44) and dorsal precentral gyrus. In the 170–250 ms time-window, however, significant interactions emerged from an ANOVA including the lobes of the reading routes (temporal and parietal cortices) along with the factors ROI (4: superior temporal, middle temporal, inferior temporal, fusiform gyrus; postcentral gyrus, supramarginal gyrus, superior parietal, inferior parietal), and group (2): these reflected that whilst ASC participants showed greater word-reading activity than controls in left parietal cortex, the latter group showed greater activity in left temporal cortex in contrast to ASC participants. This dorsal/ventral distinction between groups began to tail off in the final time-window, 375–450 ms (Fig. 2, Part E), though an interaction in left frontal lobe reflected a tendency for the ASC group to still show greater activation in more dorsal regions such as BA 44 and for control participants to show greater activation in more ventral regions.

Though there was a pattern of greater dorsal activity in the ASC group and greater ventral activity in controls, a secondary analysis focused at a within-group level and compared activation between in parietal and temporal cortices in each time-window. The control group showed greater activation of the temporal than parietal cortex for word-reading in the 170–250 ms time-window (f (1, 16) = 38.124, *p* < .001). The ASC group, in contrast, showed no significant difference between temporal and parietal cortices whilst reading.

### Semantic Category-Specificity

Semantic differences between well-matched word categories have been reported in typically-developing subjects across the range of time that we studied, beginning as early as 100 ms (Pulvermüller et al. 2001). In accordance, we investigated our dataset for category-specific group differences between action-, object- and abstract words in the time-windows previously defined. As in the previous analysis, word category effects for each time-window were explored in each lobe for each group individually, using an ANOVA employing the levels ROI (individual ROIs of each lobe: see Methods for details), Word Category (3 levels: action, object and abstract words), and hemisphere (2). Of the time-windows defined through investigation of the SNR curve for all words, category-specific differences were seen only in the 140–160 ms peak and the 170–250 ms time-windows. As previous work has illustrated that short time-windows may be best to capture focal and temporally-brief semantic differences (Pulvermüller et al. 2009), we attempted to additionally scrutinise category-specific differences in short windows 20 ms before and after the main peak (140–160 ms). Word category effects for each time-windows of interest are listed in Table 2, alongside post hoc t-tests which determined the nature of semantic effects in different brain regions.

**Table 2:** Main effects and post hoc t-tests for word categories in each group

	120–140 ms	140–160 ms	160–180 ms	170–250 ms
Control group	**Bilateral superior frontal cortex:** **f [2, 58]** **=** **7.906,** *p* < **.005** L. hemisphere: Action > object (t [16] = 2.154, *p* < .05) Action > abstract (t [16] = 2.207, *p* < .05) R. hemisphere: Action > abstract (t [16] = 2.522, *p* < .03) **Bilateral fusiform gyrus:** **f [2, 58]** **=** **5.494,** *p* < **.01** L. hemisphere: Object > abstract (t [16] = 2.460, *p* < .03) *Object* > *action* *(t [16]* = *2.060, p* < *.06)* R. hemisphere: Object > abstract (t [16] = 2.415, *p* < .03) **Bilateral ventral BA 19:** **f [2, 32]** **=** **4.702,** *p* < **.03** L. hemisphere: Object > abstract (t [16] = 2.435, *p* < .03) R. hemisphere: Object > abstract (t [16] = 2.747, *p* < .02)	**Bilateral superior frontal cortex:** **f [2, 32]** **=** **4.221,** *p* < **.03** L. hemisphere: Action > abstract (t [16] = 3.195, *p* < .01) **Bilateral BA 44:** **f [2, 32]** **=** **4.603,** *p* < **.02** R. hemisphere: Action > abstract (t [16] = 2.674, *p* < .02) **Right precentral cortex:** **f [2, 32]** **=** **4.429, p** **<** **.03** Action > object (t [16] = 2.784, *p* < .02) Action > abstract (t [16] = 2.295, *p* < .04) **Left fusiform gyrus:** **f [2, 32]** **=** **5.705,** *p* < **.01)** Object > action (t [16] = 3.084, *p* < .01) Object > abstract (t [16] = 2.718, *p* < .01) **Left BA 17:** **f [2, 32]** **=** **3.844,** *p* < **.035** Object > abstract (t [16] = 2.806, *p* < .02) **Bilateral BA 18:** **f [2, 32]** **=** **3.677,** *p* < **.04** Bilateral: Object > abstract (t [16] = 2.370, *p* < .04) **Bilateral ventral BA 19:** **f [2, 32]** **=** **4.033,** *p* < **.03** Bilateral: Object > abstract (t [16] = 2.589, *p* < .02)	**Right precentral gyrus:** **f [2, 32]** **=** **3.999, ε** **=** **.993,** *p* < **.03** Action > object (t [16] = 2.271, *p* < .04) Action > abstract (t [16] = 2.293, *p* < .04)	**Right precentral gyrus:** **f [2, 32]** **=** **4.718, ε** **=** **.796,** *p* < **.02** Action > abstract (t [16] = 2.777, *p* < .02)
ASC group	**Bilateral superior frontal gyrus:** **f [2, 26]** **=** **5.116, ε** **=** **1.000,** *p* < **.013** L. hemisphere: Object > action *(t [13]* = *2.074, p* < *.06)* R. hemisphere: Object > action (t [13] = 2.216, *p* < .05) *Object* > *abstract* *(t [13]* = *2.105, p* < *.06)*	No word category effects.	No word category effects.	**Left precentral gyrus:** **f [2, 26]** **=** **4.000,** *p* < **.04** Object > action (t [13] = 3.172, *p* < .01)

Statistical results from the analysis of different semantic word category. Main effects of word category are reported in bold font, whilst post hoc t-tests, carried out to investigate the nature of the semantic differences, are reported in standard text. Marginally non-significant results are displayed in italics

Typically-developed control participants showed a clear pattern across all time-windows where action words dominated in frontal brain regions. This was most robust in the right precentral cortex, where action words evoked greater activity than object or abstract words from 140 to 180 ms. In comparison, object words activated posterior brain regions more strongly than other word category. Most notably, t-tests showed that they evoked significantly greater activity than both action *and* abstract words in the left fusiform gyrus within the 140–160 ms time-window, though they were the dominant semantic category in all regions listed.

In comparison with the control group, very few word category effects were found for ASC participants. These were limited to effects in frontal regions which revealed a very different pattern of activity to that seen in the control group: greater activity for object words than for other word categories.

**Fig. 3:**
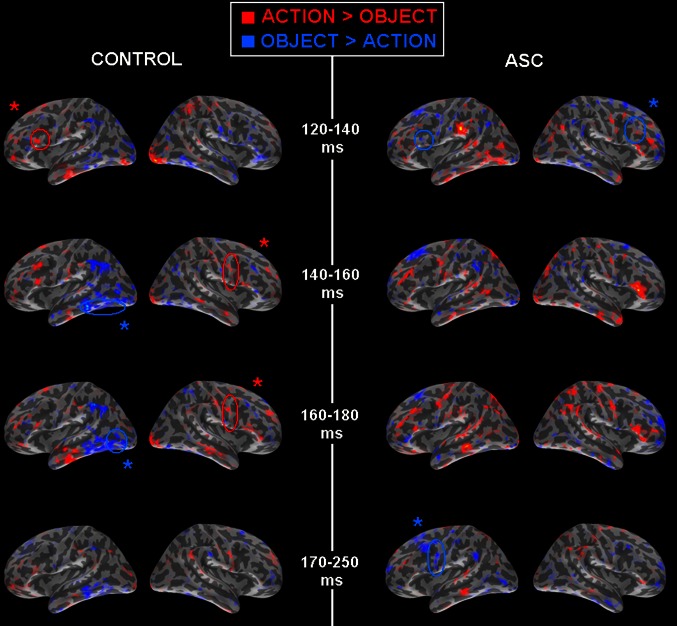
SOURCE estimations for action and object words for the control and ASC groups (*left* and* right* respectively) during each of the time-windows analysed for category-specificity. Activity in* red* reflects areas of greater activity for action than object words, whereas activity in* blue* reflects greater activity for object than action words. *Asterisks* (*) and *circles* reflect areas where within-group post hoc t-tests revealed significant differences between action and object words: *red circles* indicate significantly greater activity for action words whereas* blue circles* reflect greater activity for object words

These statistical results are reflected in Fig. 3, which displays activation maps for each group during the time-windows of interest. As the majority of literature focuses on the distinction between action- and object-related words and this is our key interest here, only this comparison is displayed (rather than comparisons with abstract words). As can be seen, many more instances of category-specificity are evident in the control group: these reflect a greater strength for object words in posterior brain regions and greater activity for action words in frontal regions. In the ASC group, the strength for object words in the 120–140 ms time-window was quite weak (as can be seen in Table 2). A stronger dominance for object words in precentral gyrus can however be seen in the 170–250 ms time-window.

## Discussion

Whilst pooled subject data in our source analysis revealed activity typical during visual word processing, further investigation of the combined EEG/EMEG dataset revealed clear group differences in several time-points of the epoch. By far the most striking observation was a pattern whereby activation for control subjects seemed to spread in a ventral fashion in contrast to the dorsal activation pattern shown by ASC participants. The latter group showed significantly greater activation than control participants in parietal regions across each of the time-windows studied. This effect did not initially discriminate between words and the control condition (70–130 ms), but from 140 ms onwards was word-specific. This suggests greater recruitment and reliance on parietal regions than that seen in the control group and implies, as in previous literature (Kamio and Toichi 2000; Toichi and Kamio 2001, 2002, 2003; Harris et al. 2006; Kana et al. 2006; Gaffrey et al. 2007), qualitatively different processing and recruitment of neural pathways in individuals with autism. Exploring differences between semantic categories revealed that control subjects showed a typical pattern of greater activity for object words in posterior temporal regions and greater activity for action words in frontal and motor systems, as has also been reported in previous research. The ASC group actually showed a reversal of this pattern, with greater activity for object than action words in bilateral superior frontal and precentral gyrus, indicating atypical representation of concepts in the brain. These were, however, the only category differences seen in this population. Previous literature has suggested that people with ASC may not automatically process words at a semantic level unless explicitly asked to do so, and our data, finding very few category effects for the ASC group in comparison to the theory-congruent pattern seen in controls, seem consistent with this proposition. These findings are discussed in more detail below.

### Reading Pathways in the Brain are Differentially Recruited in ASC

Successful reading involves flexible shifting between two pathways: a ventral, lexicosemantic pathway (left occipito-temporal cortex) engaged in direct mapping of whole-word forms to their meanings, and a dorsal, grapheme-phoneme conversion route (left parietal cortex, pars opercularis) which decodes written words in a rule-driven, piecemeal manner (Coltheart et al. 2001; Jobard et al. 2003; Levy et al. 2009). In typical readers, previous research suggests that the lexicosemantic pathway is preferentially employed in the processing of highly frequent words (Coltheart and Rastle 1994) which, being very familiar, can be matched directly and efficiently onto their lexical entries and their semantics retrieved without the necessity of prior grapheme-to-phoneme decoding of their phonological forms. The current data support this interpretation, as control subjects showed significantly greater activity in left temporal than parietal regions in the 170–250 ms time-window. At this time, word-specific activity in the left ventral route comprising of the temporal cortex was significantly greater than dorsal route activity for the control group, and significantly higher than in the ASC group as reflected by a group difference. The latter group, in contrast, showed no preferential recruitment of the lexical route or indeed of either pathway, with no significant differences between them. They showed instead an additional recruitment of the parietal cortex, significantly different to the control group, for these regularly-spelt, familiar words. Initial parietal activation by the ASC group (70–130 ms) was not specific to words—but in the same time-window, activation in dorsal precentral areas *was* word-specific for this group and also indicates utilisation of this dorsal route to inferior frontal areas.

Indeed, as the time following initial word presentation increased, this trend for dorsal activity in ASC became greater and word-specific, with greater word-elicited activation in postcentral gyrus, supramarginal gyrus, superior and inferior parietal regions in the ASC group than in controls. This trend continued to later time-windows, with greater word-specific ASC activation in left parietal cortex, left pars opercularis and dorsal precentral gyrus at 300–375 ms, and greater word-specific activation of parietal cortex at 375–450 ms. In the same period, the ASC subjects also showed greater, non-specific activity in pars opercularis (BA 44), a region also notably associated with the nonlexical route (Fiez and Petersen 1998; Fiez et al. 1999; Fiebach et al. 2002; Jobard et al. 2003), given its role in phonological processing (Paulesu et al. 1993; Fiez 1997; Poldrack et al. 1999; McDermott et al. 2003). Activity in parietal regions suggests that rather than preferentially recruiting the lexical route to map short, familiar words as whole units directly to their meanings, ASC participants perform the indirect operation of grapheme-phoneme conversion whilst reading.

This atypical recruitment of the nonlexical grapheme-phoneme conversion route whilst reading is theoretically consistent with the precocity that some autistic children show towards sounding words aloud (Newman et al. 2007), and the aforementioned relationship between ASC and hyperlexia. This suggests that the ‘mechanical’ skills of grapheme-phoneme decoding may exceed the direct mapping of letter strings to meaning. Given that phonological processing strategies play a critical role in learning to read (Rack et al. 1994), the over-reliance on this indirect phonological route which we observe here in autism is consistent with the hyperlexia sometimes observed in this population and the fact that reading problems in the literature appear to be more related to *comprehension* than to mechanical decoding and the learning process (Venter et al. 1992; Myles et al. 2002; Nation et al. 2006; Newman et al. 2007). Semantic Stroop tasks reveal that semantic processes are occurring at some level in autism (Bryson 1983; Eskes et al. 1990; Ozonoff 1997; Ozonoff and Jensen 1999; Russell et al. 1999), and it is clear that autistic individuals *can* read for meaning—but our results suggest that, in a passive task, they do not *automatically* do so in preference over the non-semantic route. Indeed, semantic-level processing may not be the ‘default mode’ of processing in ASC (Järvinen-Pasley et al. 2008) as behavioural and brain-imaging studies suggest that these individuals naturally favour perceptual processing strategies (Kamio and Toichi 2000; Toichi and Kamio 2001, 2003; Gaffrey et al. 2007), which would appear consistent with their recruitment of the phonological pathway in the present study. The convergence of electrophysiological data like this with overt behavioural processing tasks is of critical importance for future research in order to corroborate and elucidate our interpretation of these findings.

### Automatic Semantics Versus a Lack of Category-Specificity

Previous research has shown early semantic differences between word categories in the typical population that are independent of focused attention (Shtyrov et al. 2004; Pulvermüller et al. 2005). Likewise, despite not being explicitly asked to ‘read for meaning’ (only to ‘attend and read each word as it appears’), our control group showed a pattern of category-specificity whereby object words evoked greater activity in posterior temporo-occipital regions (particularly in the early time-windows) and action words evoked greater activity in frontal and motor regions throughout (particularly in the right hemisphere). This is consistent with previous literature, which has reported robust associations of visual object words with posterior temporo-occipital regions (Warburton et al. 1996; Pulvermüller et al. 1999; Martin and Chao 2001; Martin 2007) and action words with frontal motor regions (Pulvermüller et al. 2001, 2005, 2009; Hauk et al. 2004; Shtyrov et al. 2004; Tettamanti et al. 2005; Aziz-Zadeh and Damasio 2008; Hauk et al. 2008; Kemmerer et al. 2008; Boulenger et al. 2009, 2012). Such associations are suggested to arise through the formation of neural assemblies through Hebbian learning (Pulvermüller 2001), whereby object and action-related words, which are generally learnt in the presence of their real-world referent, come to evoke activity in the same regions involved in experiences with that concept in the world (e.g. executing the action or seeing/interacting with the object). The activation evoked by action words in precentral motor areas was particularly robust in our control group, persisting between 140 and 250 ms. Interestingly, this effect, though present in the left hemisphere, only reached significance in the right precentral gyrus, where greatest activity was seen for action, followed by object, then abstract words. Greater activity for action words in frontal cortex for controls was also seen in bilateral superior frontal cortex and BA 44 between 120 and 140 ms, though these frontal effects were not as long-lasting as that seen in precentral gyrus.

As can be seen in Table 2 and Fig. 3, early semantic word category effects were extremely limited in the ASC group, which might support an interpretation consistent with that given above regarding automatic access to meaning. When effects did appear they were restricted to the frontal cortex, unlike in the control group. Atypical representation of semantic categories in ASC has been suggested by previous researchers (Dunn et al. 1996; Rapin and Dunn 1997), and autistic children are known to have difficulty extrapolating shared features among category members to generate a prototype (Klinger and Dawson 2001), a process critical for typical category formation. As such, atypical representation of semantic categories is expected within this group and confirmed in the present data. Within the 120–140 ms time-window, the ASC group showed a word category effect in superior frontal cortex that was divergent in nature to that shown by the control group in the same region: greater activity for object than action words. The same trend, greater activity for object than action words in the ASC group, emerged again in the 170–250 ms time-window in left precentral gyrus. This pattern of activation is widely divergent from the activation shown in the control group, which, as previously stated, is theoretically consistent with models postulating involvement and importance of motor areas in action comprehension as well as in the encoding of action-related language (Pulvermüller 2001; Barsalou 2008; Pulvermüller and Fadiga 2010). In ASC, the lack of category-specificity for action words in frontocentral cortex, and indeed the apparent strength for object words in the same region, deviates from the norm and requires an explanation.

Whilst general abnormalities of semantic storage and processing might indeed be expected in ASC, it is possible that people with ASC show particular deviance from the norm in the processing and representation of action concepts within frontocentral motor systems. The current study lacks a behavioural test of this hypothesis, but it is suggested on the basis of structural abnormalities to cortical motor systems (Mostofsky et al. 2006) and early and pervasive motor dysfunction in ASC (Teitelbaum et al. 1998; Jansiewicz et al. 2006; Provost et al. 2007; Dewey et al. 2007; Esposito et al. 2009; Green et al. 2009). Disease or damage to motor systems is assumed to disrupt the very circuits important for action word processing (Pulvermüller and Fadiga 2010), and has been linked empirically to category-specific deficits for action words (Neininger and Pulvermüller 2001, 2003; Bak et al. 2001, 2006; Boulenger et al. 2008; Grossman et al. 2008; Bak and Chandran 2011; Kemmerer et al. 2012). Whilst the above could explain the absence of the typical action-word activation in the frontal neocortex, it leaves open the question as to why we observed greater activity for object words in these frontal regions in ASC. It is not unusual for object words to activate frontal motor systems in the typical population due to their action affordances (Carota et al. 2012). It may as such be that some elements of action semantics (such as the link between an object word and its action affordances) may be relatively preserved in ASC, whilst the semantic link between an action word and the motor system underlying that action might be especially degraded and dysfunctional. Whilst in theory this kind of action semantic information would also be jeopardised by motor dysfunction, another interpretation is that the social pragmatic nature of word stimuli was protective for object words and particularly detrimental for action words, which naturally imply an actor and often refer to social activities. Social dysfunction is at the heart of the autism (APA 2000), and so words denoting objects, which have no requirement for any social context, may still be encoded and processed in conjunction with their action referent. As the present data cannot fully confirm this hypothesis, future studies will be necessary to investigate it further.

## Conclusions

We recorded EMEG activity from high-functioning adults with ASC and IQ-matched controls whilst reading passively. Our data revealed that:

1. Whilst typical controls preferentially recruit the lexical temporal pathway for reading familiar, simple words (as opposed to the dorsal grapheme-phoneme conversion route), participants with ASC show reduced activity in this pathway;
2. Participants with ASC, unlike controls, additionally activate the dorsal parietal processing route, with no preferential difference between pathways;
3. Semantic differences between word stimuli are more limited during early processing in autism, and contrast those seen in typical controls.

These findings are consistent with previous observations which suggested that ASC participants do not utilise or access semantic information unless explicitly instructed to do so. Additional recruitment of the parietal grapheme-phoneme conversion route whilst reading is also consistent with reports of savant decoding-skills in autism.

## Electronic supplementary material

Below is the link to the electronic supplementary material.

Supplementary material 1 (DOC 400 kb)
