# Supplementary material for: Brain Routes for Reading in Adults with and without Autism: EMEG Evidence
Source: J Autism Dev Disord. 2013 Jun 9;44(1):137–53. doi: 10.1007/s10803-013-1858-z (PMC3898534; doi:10.1007/s10803-013-1858-z)
Supplement: Supplementary file 1 — Supplementary material 1 (DOC 400 kb) [file 10803_2013_1858_MOESM1_ESM.doc]

Supplementary data

Tables:

- Table 1: Psycholinguistic and semantic properties of experimental words.

Figures:

- Figure 1:

**Table 1**

|  | Action | Object | Abstract | Main effect of word type (f) |
| --- | --- | --- | --- | --- |
| Length  Bigram freq.  Trigram freq.  No. of neighbours | 4.49 (.066)  32864(1645.64)  3440.21 (345.74)  7.09 (.474) | 4.34 (.062)  37804.47 (1596.69)  4150.08 (339.51)  7.99 (.510) | 4.58 (.061)  39029.90 (1669.87)  4096.74 (436.24)  6.41 (.483) | 1.084 (p < .375)  1.873 (p <. 065)  .676 (p < .715)  1.105 (p < .360) |
| Imageability  Concreteness  Visual-relatedness  Form-relatedness  Colour-relatedness  Arousal  Valence  Action-relatedness | 4.42 (.086)  3.69 (.068)  3.84 (.110)  2.45 (.092)  1.60 (.059)  3.11 (.095)  3.82 (.092)  5.32 (.084) | 5.79 (.103)  6.22 (.068)  5.86 (.076)  3.23 (.076)  2.29 (.112)  1.40 (.056)  3.85 (.042)  2.10 (.117) | 2.59 (.116)  2.95 (.084)  2.13 (.112)  1.40 (.059)  1.21 (.053)  2.02 (.094)  3.22 (.117)  3.91 (.151) | 68.001 (p > .001)  154.326 (p > .001)  97.184 (p > .001)  47.031 (p > .001)  30.960 (p > .001)  28.800 (p > .001)  18.244 (p > .001)  78.321 (p > .001) |

Table 1: Mean sycholinguistic and semantic features of word stimuli groups (standard error in brackets). Statistical tests between action and object words are displayed in *t* values.

**Figure 1**


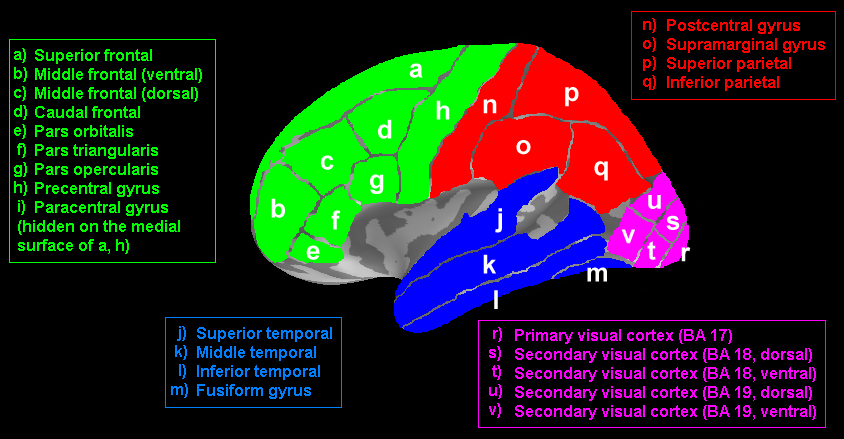


Figure S1: Anatomical ROI divisions based on the Desikan-Killiany Atlas subdivisions of the brain (Desikan et al, 2006) as implemented in the Freesurfer package. The frontal lobe was subdivided into 9 regions (in green); the temporal lobe 4 regions (in blue); the parietal lobe 4 regions (red); and the occipital lobe into 5 regions (pink).
